# 

**Published:** 1896-04

**Authors:** 


### CONTENTS.

COMMUNICATIONS.

The Destruction of Children's Teeth—Cause and Prevention .......... 157
Common Ground of Medicine and Dentistry .......... 161
New Treatment for Pyorrhœa Alveolaris .......... 165

SELECTIONS.

The Turkish Bath .......... 167
A Long-Lived Family .......... 169
Classification of Diseases .......... 169
Infective Inflammation of the Parotid Glands .......... 170
Deodorization of Iodoform .......... 172
Specific Treatment of Necrosis of the Alveoli and Maxillæ with Aromatic Sulphuric Acid .......... 173
A New Picture Process .......... 177
A Slight Misunderstanding .......... 178
"Ter Die" .......... 178
The Use of Vinegar to Prevent Vomiting after Chloroform Inhalation .......... 179
The Influence of Mind .......... 179
Limewater .......... 180
Aconite to Abort Colds .......... 180
The St. Petersburg School of Medicine for Women .......... 181
Painless Tooth Extraction .......... 181
Antiseptics .......... 181
Asbestos .......... 181
Is the Bacteriological Theory of Disease Waning? .......... 182
A Slap at the Bacteriologists .......... 183
Cheerfulness .......... 184
Possibilities of the X-Rays in Surgery .......... 185
Electrical Effects of Sprays .......... 187
The Treatment of Burns .......... 188
Camphor for Ether Collapse .......... 189
Insomnia .......... 189
Chlorate of Potash as a Tooth Powder .......... 190
Medical Students in Paris .......... 190
The Medical Cuckoo .......... 191
The Influence of Odors upon the Voice .......... 192
Face Reading .......... 192
The Metric System .......... 193
What is a Poison? .......... 194
Hardening Steel by Gas .......... 195
What Becomes of the Microbes? .......... 196
Decline of Alcohol as a Medicine .......... 197
Alcohol as a Panacea .......... 199
A Report of the Committee on the Abuse of Alcoholic Drinks from a Sanitary Standpoint .......... 200
Discussion of a Paper on "The Dentist in his Profession Among the People" 200

EDITORIAL.

Alabama State Dental Association .......... 205
The Editorial "We" .......... 206
American Medical Association—Dental Section .......... 206
Bibliographical .......... 207
Dental Chemistry and Metallurgy .......... 207
Obituary .......... 208

